# Semi-invasive pulmonary aspergillosis in an immunosuppressed patient: a case report

**DOI:** 10.1186/1757-1626-2-40

**Published:** 2009-01-12

**Authors:** Fernanda C Cabral, Edson Marchiori, Gláucia Zanetti, Tatiana C Takayassu, Claudia M Mano

**Affiliations:** 1 Department of Radiology of the Federal University of Rio de Janeiro, Rua Professor Rodolpho Paulo Rocco, 255, Cidade Universitária, CEP 21941-913, Rio de Janeiro, Brazil; 2 Department of Radiology of the Fluminense Federal University, Rua Marquês do Paraná, 530. Centro, CEP 24000-000 Niterói, Rio de Janeiro, Brazil

## Abstract

**Background:**

The authors present the high-resolution computed tomography findings of an immunosuppressed patient with semi-invasive pulmonary aspergillosis.

**Case presentation:**

The main finding consisted of irregular, thick-walled cavity in the right upper lobe and a mass with soft-tissue attenuation within it and thickening of adjacent pleura. Additional findings were bronchial wall thickening associated with a 'tree-in-bud' pattern. Following the clinical, laboratorial and imaging criteria, the diagnosis of semi-invasive pulmonary aspergillosis was defined and antifungical treatment was introduced.

**Conclusion:**

The patient responded well to the treatment with improvement in his systemic symptoms and regression of the pulmonary lesions.

## Background

*Aspergillus *is a saprophytic, aerobic fungus that develops on dead or decaying organic matter and produces airborne spores that can be inhaled by man [1]. The pulmonary aspergillosis manifestations are determined by the patient's immune status and the presence of underlying disease [2].

Pulmonary aspergilosis can be subdivided into five categories: saprophytic aspergillosis (aspergiloma), hypersensitivity reaction (allergic bronchopulmonary aspergillosis), semi-invasive (chronic necrotizing) aspergillosis, airway-invasive aspergillosis (acute tracheobronchitis, bronchiolitis, bronchopneumonia, obstructing bronchopulmonary aspergillosis), and angioinvasive aspergillosis [2]. Semi-invasive aspergillosis, also known as chronic necrotizing aspergillosis (CPNA) is a chronic form, in which the fungus is intermediate between a simple saprophyte and an invasive pathogen [3]. This condition occurs predominantly in patients with some evidence of mild systemic immunodeficiency such as chronic debilitating illness, immunosuppression therapy or advanced age. Furthermore, the presence of superimposed structural lung disease, such as chronic obstructive pulmonary disease or bronchiectasis adds an additional risk factor [3-6]. The purpose of this report is to present the radiographic and computed tomography (CT) findings of semi-invasive aspergillosis in a lupic and diabetic patient who underwent renal transplant and subsequent immunosuppression therapy.

## Case presentation

A 24-year-old black man, presented with an one-month history of productive cough, weight loss and malaise. He was diagnosed to have systemic lupus erythematosus 6 years ago and he had pulmonary tuberculosis 4 years ago and for this he received one year of antituberculosis treatment. Furthermore he underwent renal transplant 3 years ago, and developed post-transplant diabetes mellitus. He was receiving prednisone, tacrolimus and rapamicine. He had never smoked and there was no history of alcohol abuse.

Physical examination showed the patient to be ill-looking, emaciated, acianotic, and afebrile. Blood pressure of 100/70 mmHg, pulse rate of 80 beats per minute and respiratory rate of 22 breaths per minute. Laboratory tests showed the following: hemoglobin- 9,60 g/dl, hematocrit- 30,4%, leukocytes- 8200/cu mm (49% segmented neutrophils, band- 2%), lymphocytes- 22%. Prothrombin time were 50% (16"1 versus 11"5 control value), International Normalized Ratio (INR) 1,40, partial thromboplastin time (relation patient/control) were 1,50, albumin of 1,0 g/dl, urea of 99 mg/dl and creatinine of 3 mg/dl. HIV test and Ziehl Neelsen stain for acid-fast bacili were negative. Bronchoscopy showed no abnormalities. *Aspergillus fumigatus *was isolated by cultures of sputum and bronchoalveolar lavage. Immunodiffusion tests showed no *Criptoccocus, Histoplasma *or *Aspergillus *on blood. A pulmonary biopsy was not indicated because of his hypoproteinemia and coagulation disturb.

**Figure 1:**
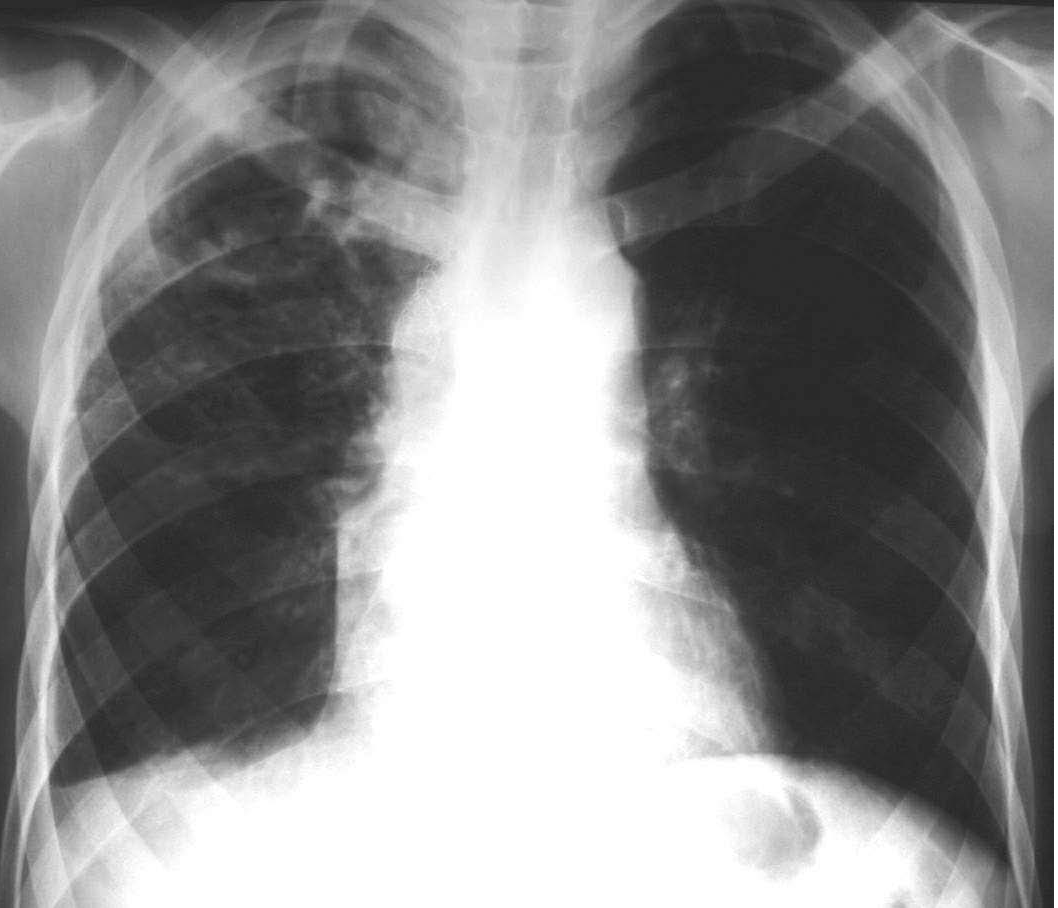
**Chest radiograph (postero-anterior view) showing an irregular, thick-walled cavity in the right upper lobe**. Note also the pleural reaction in this hemithorax.

**Figure 2:**
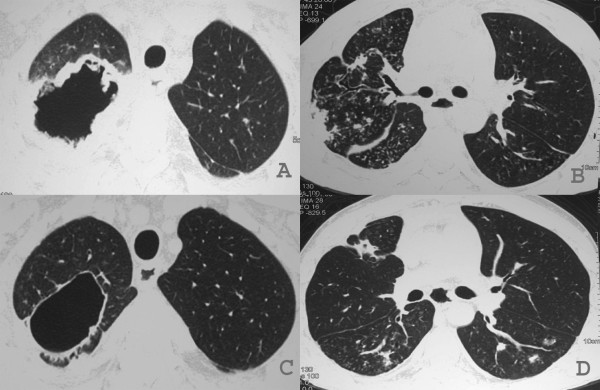
**(A) Computed tomography (CT) scan at level of upper lobes showing a thick-walled cavity with irregularity of its inner contour in the right upper lobe**. (B) CT scan at the subcarinal level, showing air-space nodules, tree-in-bud pattern and consolidation at the periphery of the right lung. Fissural thickening and bronchial wall thickening and irregularity. Observe also small nodules in the left lung. (C and D) CT scans obtained at the same level as (A and B) two months after showing substantial improvement of the appearance of the lesions, persisting a thin-walled cavity in the right upper lobe and scattered small nodules in the lungs.

The chest radiograph revealed a thick-walled cavity in the right upper lobe with adjacent pleural thickening (Figure 1). The high-resolution computed tomography (HRCT) showed an irregular, thick-walled cavity in the right upper lobe and a mass with soft-tissue attenuation within it and thickening of adjacent pleura. Additional findings were bronchial wall thickening associated with a 'tree-in-bud' pattern, characterizing bronchiolitis, in the right upper, middle and lower lobes, and in the left lower lobe (Figure 2A and 2B). Along with this, a small bilateral pleural effusion was seen.

He received empirical treatment for tuberculosis during one month with no clinical response. Failure to respond to therapy and associating the radiologic, laboratory, and clinical findings a diagnostic of semi-invasive aspergillosis was made, so he was started on oral voriconazole 200 mg once every 12 hours. The patient responded well to the treatment with improvement in his systemic symptoms and regression of the pulmonary lesions (Figure 2C and 2D).

## Discussion

CPNA is a rare locally destructive form of aspergillosis [7]. The species found most commonly in CPNA is *A. fumigatus *[3,5,8,9], which was the fungi isolated in this case. At histological analysis, this disease is characterized by the presence of tissue necrosis and granulomatous inflammation [2]. The development of this form of aspergillosis depends largely on the immune status of the patient and the presence of superimposed structural lung disease; so risk factors include: diabetes mellitus, chronic debilitating illness, alcoholism, advanced age, prolonged corticosteroid therapy, chronic obstructive pulmonary disease, sarcoidosis, a history of pulmonary tuberculosis, previous resectional surgery, pneumoconiosis, collagen vascular disease, previous radiation therapy and malnutrition. Furthermore patients with chronic airflow limitation, who are taking long term oral corticosteroids in conjunction with recurrent courses of systemic antibiotics are also at risk [1,4,7,10,11]. In the present case, a lupic, diabetic patient who had a history of tuberculosis was taking immusuppressor after renal transplantation.

The typical time course of CPNA extends over a period of several months to years, and the clinical symptoms include chronic cough, sputum production, fever and constitutional symptoms such as weight loss and weakness. Hemoptysis is seen in only 15% of patients [2,3,9,10]. It is often difficult to obtain diagnostic confirmation of CPNA by histologic evidence of local lung tissue invasion by septal hyphae, consistent with *Aspergillus*. Therefore other diagnostic criteria have been proposed. Soubani e Chandrasekar [12] have proposed the following criteria: clinical and radiologic features consistent with the diagnosis; isolation of *Aspergillus *species by culture from sputum or from bronchoscopic or percutaneous samples; and exclusion of other conditions with similar presentation, such as active tuberculosis, atypical mycobacterial infection, chronic cavitary histoplasmosis, or coccidioidomycosis. The slow progression of clinical and radiographic findings may contribute to a delay in diagnosis [9]. In this report, the diagnosis of CPNA was made on the basis of clinical findings such as cough, sputum production and constitutional symptoms associated with radiographic appearance consistent with CPNA and the isolation of *Aspergillus *from cultures of sputum and bronchoalveolar lavage.

Radiological features include multiple nodular opacities and areas of consolidation with or without cavitation or adjacent pleural thickening [3,9]. It usually occurs in the upper lobes [8]. Cavity formation is a manifestation of the invasiveness of the fungus and does not reflect colonisation of a preexisting cavity, distinguishing CPNA from aspergilloma [5,11]. The cavities that develop in CPNA frequently contain ball like collections similar to aspergillomas, but is preferable to use the term [11]. Spread may be to the entire lung, chest wall or mediastinum [8]. Radiological findings in this case consisted of an irregular, thick-walled cavity in the right upper lobe, a mass with soft-tissue attenuation within it, thickening of adjacent pleura and bronchial wall thickening associated with a 'tree-in-bud' pattern.

Treatment outcome is likely to be influenced by the rapidity of the therapy beginning. Amphotericin B and itraconazole have been used with success in several cases of CPNA, however new azole antifungal drugs such as voriconazole, posaconazole and ravuconazole have a broad spectrum of activity and are an alternative to *Aspergillus *species resistant to amphotericin B [3,13,14]. Voriconazole was the treatment of choice because it can be orally administered and it has been used with success in some series of patients. Surgical resection is reserved for patients with hemoptysis, for those who are not tolerating antifungal agents, and patients with residual localised but active disease despite adequate antifungal therapy [5,10,15].

## Competing interests

The authors declare that they have no competing interests.

## Authors' contributions

FCC conceived the study. FCC, EM and GZ research the literature review and prepared the manuscript. FCC, EM and TCT edit and coordinated the manuscript. All authors read and approved the final manuscript.

## Consent

Written informed consent was obtained from the patient for publication of this case report and accompanying images. A copy of the written consent is available for review by the Editor-in-Chief of this journal.

## References

[B1] Soto-Hurtado EJ Marin-Gamez E Segura-Dominguez N Jimenez-Onate F Pleural aspergillosis with bronchopleurocutaneous fistula and costal bone destruction: a case report Lung 2005 183 417 423 10.1007/s00408-005-2553-4 16465601

[B2] Franquet T Muller NL Gimenez A Guembe P de La Torre J Bague S Spectrum of pulmonary aspergillosis: histologic, clinical, and radiologic findings Radiographics 2001 21 825 837 11452056 10.1148/radiographics.21.4.g01jl03825

[B3] Gefter WB Weingrad TR Epstein DM Ochs RH Miller WT "Semi-invasive" pulmonary aspergillosis: a new look at the spectrum of aspergillus infections of the lung Radiology 1981 140 313 321 7255704 10.1148/radiology.140.2.7255704

[B4] Binder RE Faling LJ Pugatch RD Mahasaen C Snider GL Chronic necrotizing pulmonary aspergillosis: a discrete clinical entity Medicine (Baltimore) 1982 61 109 124 10.1097/00005792-198203000-00005 7038373

[B5] Gefter WB The spectrum of pulmonary aspergillosis J Thorac Imaging 1992 7 56 74 1404546 10.1097/00005382-199209000-00009

[B6] George PJ Boffa PB Naylor CP Higenbottam TW Necrotising pulmonary aspergillosis complicating the management of patients with obstructive airways disease Thorax 1983 38 478 480 6879504 10.1136/thx.38.6.478 PMC459590

[B7] Parakh UK Sinha R Bhatnagar AK Singh P Chronic necrotising pulmonary aspergillosis: a rare complication in a case of silicosis Indian J Chest Dis Allied Sci 2005 47 199 203 16022149

[B8] Aquino SL Kee ST Warnock ML Gamsu G Pulmonary aspergillosis: imaging findings with pathologic correlation AJR Am J Roentgenol 1994 163 811 815 8092014 10.2214/ajr.163.4.8092014

[B9] Franquet T Muller NL Gimenez A Domingo P Plaza V Bordes R Semiinvasive pulmonary aspergillosis in chronic obstructive pulmonary disease: radiologic and pathologic findings in nine patients AJR Am J Roentgenol 2000 174 51 56 10628453 10.2214/ajr.174.1.1740051

[B10] Thompson BH Stanford W Galvin JR Kurihara Y Varied radiologic appearances of pulmonary aspergillosis Radiographics 1995 15 1273 1284 8577955 10.1148/radiographics.15.6.8577955

[B11] Wiggins J Clark TJ Corrin B Chronic necrotising pneumonia caused by Aspergillus niger Thorax 1989 44 440 441 2763249 10.1136/thx.44.5.440 PMC461860

[B12] Soubani AO Chandrasekar PH The clinical spectrum of pulmonary aspergillosis Chest 2002 121 1988 1999 10.1378/chest.121.6.1988 12065367

[B13] Fratti RA Belanger PH Sanati H Ghannoum MA The effect of the new triazole, voriconazole (UK-109,496), on the interactions of Candida albicans and Candida krusei with endothelial cells J Chemother 1998 10 7 16 9531069 10.1179/joc.1998.10.1.7

[B14] Saraceno JL Phelps DT Ferro TJ Futerfas R Schwartz DB Chronic necrotizing pulmonary aspergillosis: approach to management Chest 1997 112 541 548 10.1378/chest.112.2.541 9266898

[B15] Kim JS Rhee Y Kang SM Ko WK Kim YS Lee JG Park JM Kim SK Lee WY Chang J A case of endobronchial aspergilloma Yonsei Med J 2000 41 422 425 10957902 10.3349/ymj.2000.41.3.422

